# A Framework for Disseminating Evidence-Based Health Promotion Practices

**Published:** 2011-12-15

**Authors:** Jeffrey R. Harris, Allen Cheadle, Peggy A. Hannon, Patricia Lichiello, Mark Forehand, Eustacia Mahoney, Susan Snyder, Judith Yarrow

**Affiliations:** University of Washington, Health Promotion Research Center, Seattle, Washington; University of Washington, Health Promotion Research Center, Seattle, Washington; University of Washington, Health Promotion Research Center, Seattle, Washington; University of Washington, Health Promotion Research Center, Seattle, Washington; Michael G. Foster School of Business, University of Washington, Seattle, Washington; American Cancer Society, Seattle, Washington; Susan Snyder, Senior Services, Seattle, Washington; Senior Services, Seattle, Washington; University of Washington School of Public Health, HPRC

## Abstract

Wider adoption of evidence-based, health promotion practices depends on developing and testing effective dissemination approaches. To assist in developing these approaches, we created a practical framework drawn from the literature on dissemination and our experiences disseminating evidence-based practices. The main elements of our framework are 1) a close partnership between researchers and a disseminating organization that takes ownership of the dissemination process and 2) use of social marketing principles to work closely with potential user organizations. We present 2 examples illustrating the framework: EnhanceFitness, for physical activity among older adults, and American Cancer Society Workplace Solutions, for chronic disease prevention among workers. We also discuss 7 practical roles that researchers play in dissemination and related research: sorting through the evidence, conducting formative research, assessing readiness of user organizations, balancing fidelity and reinvention, monitoring and evaluating, influencing the outer context, and testing dissemination approaches.

## Introduction

Although the public health community has developed many evidence-based practices to promote healthy behaviors, adoption of these practices has been haphazard (1,2). In response, the Centers for Disease Control and Prevention (CDC) and the National Institutes of Health (NIH) have called for more attention to dissemination of evidence-based practices and for research on how to increase dissemination effectiveness (3-5).

Several conceptual frameworks have been developed to organize the extensive literature on diffusion and dissemination of evidence-based practices. Particularly relevant for the dissemination of evidence-based health promotion practices are those of Greenhalgh et al (6) and Wandersman et al (7). Greenhalgh, focusing on system-level practices in large health care organizations, reviewed the literature on dissemination and diffusion and developed a conceptual framework to organize it. Wandersman focused more directly on health promotion practices that might be implemented in both small and large organizations; his interactive systems framework (ISF) highlights the roles of key actors in the dissemination process. Another recently developed framework that synthesizes several existing frameworks (including that of Greenhalgh) is the consolidated framework for implementation research (CFIR) (8). Finally, the RE-AIM framework, though developed for evaluation, is widely used to provide organizing principles for the dissemination of evidence-based practices (9).

These frameworks are useful for generating hypotheses for future research, but no practical framework exists for developing and testing dissemination approaches. Such a framework would serve as a guide to dissemination for community-based organizations and help researchers develop and test approaches to dissemination of evidence-based practices.

We describe a practical framework for dissemination developed at the University of Washington Health Promotion Research Center (HPRC), a Prevention Research Center funded by CDC to conduct research on community-based prevention and control of chronic diseases. To illustrate the framework, we use 2 dissemination approaches we have developed and tested and discuss practical roles researchers play in dissemination and dissemination research.

## HPRC Dissemination Framework

The evidence-based practices that can be disseminated using our proposed framework (Figure) include environmental changes, policies, programs, and systems. Examples of evidence-based practices include research-tested environmental changes (eg, increased availability of healthy food in schools and workplaces), policies (eg, employer insurance coverage of treatment for tobacco cessation), programs (eg, healthy-aging exercise programs, such as Matter of Balance [10] and Active for Life [11]), and systems change (eg, expanded hours for mammography clinics).

**Figure.**
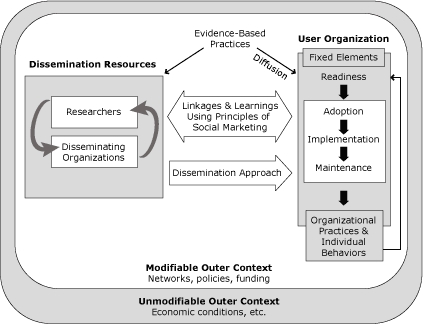
The dissemination framework shows the resources (researchers and disseminating organizations) affecting a user organization through a dissemination approach developed collaboratively, using social marketing principles. The framework functions in an outer context of modifiable and unmodifiable elements.

**Table:** 

The HPRC dissemination framework has 2 key elements: the resources for disseminating an evidence-based practice, which include collaborating researchers and disseminating organizations, and the user organization that adopts and implements the practice. The dissemination approach is developed by using social marketing principles and requires linking to and learning from the user organization to develop relevant and effective dissemination. The user organization has characteristics that affect its readiness to change. Each characteristic is dependent on the preceding one. These characteristics start with the fixed elements and the readiness to adopt an intervention. Readiness affects the 3 stages of adoption (adoption, implementation, and maintenance). Finally, the implementation of intervention affects the organization's practices and individuals' behaviors, which in turn feeds back into the organization by affecting its fixed elements and readiness to change. The framework also acknowledges that the adoption of an intervention may also occur through passive diffusion. The elements of the framework — the resources, user organization, and dissemination approach — exist in an outer context of modifiable (networks, policies, funding) and unmodifiable (economic conditions, etc.) elements.
This framework is based on existing literature on dissemination, including Greenhalgh (5), Wandersman's interactive systems framework (6), the consolidated framework for implementation research (7), and RE-AIM (8), as well as our experience disseminating evidence-based practices.

The HPRC framework acknowledges that practices can spread either passively or actively. The "diffusion" arrow illustrates that some evidence-based practices spread passively and are adopted without additional support or instigation from outside the user organization (12). The larger dissemination approach arrow shows that diffusion alone is often not an effective way of spreading evidence-based practices and that specific efforts are required to encourage widespread implementation. The framework focuses on the active dissemination process.

The HPRC framework has 3 main actors: researchers and disseminating organizations (Figure, left) and user organizations (Figure, right). Researchers seek to create new knowledge to aid dissemination of best practices. Disseminating organizations ("disseminators") use that knowledge to lead dissemination efforts. User organizations put best practices into place. Disseminators may be nonprofit organizations that market an evidence-based practice (eg, through licensing or sales of a branded health promotion program) or foundations or governmental agencies that fund user organizations to support adoption and researchers to evaluate implementation of evidence-based practices (eg, grant funding provided by the Administration on Aging to encourage adoption of evidence-based practices).

Researchers and disseminators build a mutually beneficial collaborative partnership. The partnership can be initiated by either party and, in our experience, is most effective when the research team and the disseminator are trying to reach the same user organizations and are willing to learn about each other's resources and goals. Building on this learning, both the researchers and the disseminator are better equipped to design and test dissemination approaches that fit the disseminator's goals and capacity. Researchers work closely with disseminators in refining and testing the dissemination approach to make it more suitable for user organizations. Although researchers can serve as disseminators, disseminators have at least 2 advantages in disseminating best practices: 1) they can focus on dissemination, rather than on research objectives and funding, and thus focus on the support systems needed to reach the scale necessary to make a difference at a population level; and 2) they may be closer in culture and values to user organizations than are researchers and thus better able to promote best practices and adapt them to local needs.

Successful dissemination of evidence-based practices in a user organization involves a cascade of steps (Figure). Steps include adoption, implementation, and maintenance. The cascade also acknowledges the fixed elements of the user organization — that is, the state of the organization before dissemination — and its readiness to support adoption, implementation, and maintenance, including the availability of human and financial resources. The terminology parallels the adoption, implementation, and maintenance steps in the RE-AIM framework (9). The output of this cascade is change in organizational practices and personal behaviors that result in improved health and other benefits (eg, increased productivity) for either the members of the organization (eg, its workers) or the consumers it serves.

Between the boxes showing the researchers and the disseminators on the left and the user organizations on the right is the bi-directional "linkages and learnings" arrow. This arrow highlights the need for understanding user organizations and all potential steps in the implementation process, from readiness factors through how to motivate adoption and facilitate implementation and maintenance. We believe these linkages and learnings should be informed by principles of social marketing, which focus on the needs and capabilities of user organizations. Applying these principles, the disseminator begins with a market analysis (13). This analysis assesses the potential benefits of the evidence-based practices — to both the user organization and to its members and consumers targeted for behavior change — and potential barriers to adoption (both organizational and individual). The analysis evaluates 5 key areas (13):

- Consumers: the needs of the organizational and individual *consumers* of the practice(s).
- Competition: the salient alternatives that serve as *competition* for the practice(s).
- Company: the capacity of the *company* (in our framework, the combined capacity of the researchers and disseminator) to support dissemination of the new practice(s).
- Collaborators: the strengths of *collaborators* — potential support networks or other partners — that can offer input on the constraints of real-world settings and facilitate dissemination in much the same way that a retailer helps distribute products to consumers.
- Context: the sociopolitical *context*, described as the modifiable and unmodifiable "outer context" in our framework. Modifiable context — such as organizational networks, funding, and policy support (eg, reimbursement for program participation) — is within the sphere of influence of the disseminators and researchers. Unmodifiable context includes the overall economic and political climate.

By conducting a full market analysis, the disseminator will be prepared to decide which segments of potential user organizations to target and how to best position the evidence-based practice(s) for these targeted segments (14). The process of *segmentation* can begin either at the organizational level (identifying unique groups of user organizations that are particularly well-equipped to adopt the practice) or at the individual level (identifying unique groups of individuals that the disseminator believes should adopt the behavior(s) that the practice promotes). In *targeting*, the disseminator identifies the specific segment(s) for which it wishes to develop a dissemination approach. Once the target is identified, the dissemination approach should be *positioned* to communicate the benefits of the promoted practices clearly and concisely, stressing the particular benefits that are expected to resonate with the target segment. At the level of the user organization, for example, positioning asks, What specific organizational needs will be met with this practice, more so than with other practices, and why?

In summary, the HPRC framework proposes that to disseminate evidence-based practices effectively, researchers must 1) collaborate with a disseminator and 2) work with user organizations to refine the practice and approach to dissemination, guided by the principles of social marketing.

## Two Dissemination Examples

**Box 1.** Health Promotion Research Center Dissemination Case Study Examples

**Framework Construct**	**EnhanceFitness (EF)**	**American Cancer Society (ACS) Workplace Solutions (WPS)**
Evidence-based practice	Older adult exercise program, involving aerobic activity and training to increase balance, flexibility, and strength, 3 times/wk.	Package of 15 evidence-based workplace health promotion practices to improve workers' cancer screening, nutrition, physical activity, tobacco cessation, and weight management.
Disseminating organization	Senior Services: partner on original EF efficacy research.	American Cancer Society (ACS) Great West Division: partner on original development and research; ACS National Home Office: disseminator.
User organizations	Senior centers, community centers, and nonprofit and for-profit fitness organizations (eg, the YMCA).	Employers
Linkages and learnings: working with user organizations to refine practice, dissemination approach	Developed and tested chair-based version of EF for frail older adults. Developed an online version of EF instructor training to reduce costs associated with training and to reach a wide instructor pool. Tested EnhanceMobility; an adaptation of EF for people with dementia.	Surveyed employers in target community of mid-sized employers in low-wage industries to assess their needs and resources. Interviewed ACS staff members to assess their readiness and training needs to support employers using ACS WPS.
Dissemination approach	Senior Services licenses program and provides training, materials, and data management and analysis.	ACS staff act as change agents to disseminate to employers across the United States.
Modifiable context: Policy supports	Acquired CDC Arthritis Program approval of EF. Acquired approval for EF to be 1 of 5 evidence-based disease prevention programs included in the Administration on Aging (AoA) Choices for Independence grants, 2009.	Worked to pass state laws that mandate workplace no-smoking policies as recommended by ACS WPS. Worked to pass state-level legislative mandates for insurance for colorectal cancer screening as recommended by ACS WPS.
Modifiable context: funding supports	Selected as a Choices program for AoA funding (26 states). Part of a $7.5 million initiative in South Florida.	Worked to increase state-level funding for tobacco-cessation quitlines for low-income, uninsured workers. Combined ACS WPS with another ACS workplace practice supported by state governments in Colorado, New Mexico, and Washington.
Dissemination results to date	As of 2010, offered at 523 community locations in 32 states, and has served 23,241 participants; 10,282 in 2010.	As of 2010, 1,385 employers had participated in ACS WPS, reaching more than 2.5 million workers.

**Box 2.** Researcher Roles and Contributions in Disseminating Evidence-Based Practices

**Role**	**Context**	**Researcher Contribution**
Sorting through evidence	Systematic reviews exist (eg, *Guide to Community Preventive Services* [18]; Cochrane Collaboration reviews [http://www.cochrane.org/]).	Provide an important service to disseminators and user organizations, such as summaries of reviews and customized literature reviews.
Conducting formative research	Effective dissemination depends on formative research about disseminators, user organizations, and people who are targets for health behavior change.	Assist with qualitative and quantitative research to ensure that dissemination approaches are appropriate for their intended organizational and individual audiences.
Assessing readiness of user organizations	Dissemination resources are limited; some user organizations will be more ready to adopt and implement evidence-based practices than others.	Develop readiness assessments that disseminators can use to channel resources to user organizations most likely to take advantage of them.
Balancing fidelity and reinvention	Tension often exists between fidelity of implementation necessary to ensure practice effectiveness and practice reinvention necessary to allow local ownership.	Identify *core* elements of the practice that must be implemented with fidelity to ensure effectiveness and *modifiable* elements of the practice that can be reinvented to fit local needs and context.
Monitoring and evaluating	User organizations need tools for rapid-cycle monitoring and evaluation of implementation success.	Help develop monitoring and evaluation tools.
Influencing outer context	User organizations often have less access to or credibility with policy makers than do researchers.	Influence policy and other changes needed to facilitate dissemination, through role as technical experts.
Testing dissemination approaches	If the field of dissemination is to move forward, dissemination approaches must undergo formal testing.	Conduct formal tests of dissemination approaches; obtain funding to support such tests.

In this section, we present 2 examples to illustrate the dissemination framework (Box 1). EnhanceFitness (EF) promotes physical activity among older adults, and American Cancer Society (ACS) Workplace Solutions (WPS) promotes the prevention of chronic disease among workers. In each example, we italicize key terms from Box 1.

### EnhanceFitness

EF, an *evidence-based practice*, is a low-cost, highly adaptable, group exercise program for older adults. EF was originally designed and tested by a research partnership that included the Northshore Senior Center in Bothell, Washington; Group Health Cooperative, a health maintenance organization; and HPRC. In its original trial, EF increased physical activity among participants and helped them to maintain a higher level of functioning than that of the comparison population (15).

For more than a decade, Senior Services, a nonprofit community-based organization in King County, Washington, has served as the *disseminating organization* for EF (16). For several years, HPRC and Senior Services have used *linkages and  learnings* to refine and adapt EF to meet the needs of *user organizations*, including senior centers, and special populations, such as frail older adults and those with dementia. As its *dissemination approach*, Senior Services licenses EF to senior centers and other user organizations, which pay a licensing fee, and uses the revenue from licensing to aid implementation by these user organizations. Supported by the licensing fee, Senior Services offers instructor certification classes for EF at sites around the country, provides technical assistance,training, and marketing materials, and collects and analyzes program fidelity and fitness data.

To *modify the outer context* to increase the recognition and credibility of EF, Senior Services and HPRC have obtained approval of EF from national organizations, including the CDC Arthritis Program and the Administration on Aging. HPRC has also partnered in local funding initiatives that support EF. *Dissemination results* to date show that EF reaches diverse participants nationwide. Of more than 23,000 participants, 28% are members of racial/ethnic minorities, 38% are older than age 80, 60% of those reporting income have low or very low income, 2% speak limited or no English, and 4% are immigrants or refugees.

### American Cancer Society Workplace Solutions

ACS WPS offers employers a package of *evidence-based practices* aimed at promoting health in the workplace and preventing chronic diseases among workers. ACS's Great West Division and HPRC jointly developed ACS WPS and then conducted a pilot test with 8 large employers in the Pacific Northwest (17). The pilot test showed a significant increase in these employers' implementation of the ACS WPS practices. After the pilot study, the ACS National Home Office assumed the role of national *disseminating organization* of ACS WPS. Using *linkages and learnings*, HPRC and ACS are refining and adapting ACS WPS to meet the needs of employers, the principal *user organizations*. HPRC and ACS work with a range of employers, including small and mid-sized employers and those in low-wage industries. As its *dissemination approach*, ACS deploys local and regional staff who act as change agents (external technical experts working inside a user organization) (2). After a formal assessment of the employers' implementation of evidence-based workplace health promotion practices, ACS staff conduct a gap analysis and present employers with information and technical assistance aimed at increasing adoption and implementation of recommended practices. ACS has trained approximately 15% of its staff nationwide in the principles and processes supporting ACS WPS and is identifying ways to modify ACS WPS to increase its effectiveness and make it sustainable for ACS staff. To *modify the outer context*, ACS and HPRC have worked to pass state laws and funding that support practices recommended by ACS WPS. They have also adapted another of ACS's state-funded workplace health promotion packages to include ACS WPS principles and a subset of its practices. *Dissemination results* to date show that ACS WPS reaches diverse employers nationwide. HPRC and ACS continue to measure the effect of ACS WPS on workplaces' implementation of recommended practices.

## Discussion

The HPRC framework for dissemination and dissemination research builds on the literature and has been shaped by our successful experience in developing and disseminating chronic disease prevention practices. As the 2 examples show, we have worked with private organizations as national disseminators, using theory-based dissemination approaches, and have achieved nationwide reach of evidence-based practices. The Workplace Solutions package of evidence-based practices includes environmental changes, policies, programs, and systems changes, so we believe this framework is applicable to all of these areas and to the broad field of translational research (5,18).

### HPRC framework and existing literature on dissemination

The HPRC framework incorporates elements from the Greenhalgh framework and parallels key elements of the Wandersman (ISF) framework; each of these frameworks is based on a review of a large body of literature. The key dissemination resources in our framework closely parallel those in Greenhalgh's resource system, namely the key roles for a change agency ("disseminating organization" in our terminology) and "knowledge purveyors" (researchers). Also from Greenhalgh, we adopt the idea that the process of implementation in an organization is organization-specific and involves a complex series of steps. Researchers, therefore, need to work closely with both disseminators and user organizations in designing dissemination approaches (Figure).

Systems in the Wandersman ISF framework also parallel key elements of the HPRC framework. The ISF Prevention Support System, which assists in the process of dissemination, corresponds to our "dissemination resources" (7). And our process of researchers working with the disseminators and user organizations to refine the practice and dissemination strategy is a close approximation of the ISF Prevention Synthesis and Translation System.

Kreuter and Bernhardt (19) highlight an additional need for effective dissemination of public health products — marketing and distribution systems. For commercial products, these systems include transfer to distributors, distribution to consumer outlets, inventory management, sales, technical assistance, customer service, and repair. In our framework, we imply the need for marketing and distribution systems in our use of marketing principles, but these systems deserve special attention from disseminators. Although some nonprofit health agencies serving as disseminators have marketing and distribution systems at a national scale (eg, AARP, ACS, and the YMCA), most do not and may need to work with commercial partners to reach the scale needed to make a difference at a population level.

### Roles for researchers

In the HPRC framework, researchers and disseminators form a partnership to enhance their ability to spread evidence-based practices. The contribution of researchers to the partnership includes 7 practical roles (Box 2): 1) sorting through the evidence, 2) conducting formative research, 3) assessing readiness of user organizations, 4) balancing fidelity and reinvention, 5) monitoring and evaluating, 6) influencing the outer context, and 7) testing dissemination approaches. Box 2 outlines the context in which these roles apply and the related contributions researchers can make.

To maximize real-world applicability of dissemination approaches, researchers should test them in collaboration with disseminators. When testing dissemination approaches, researchers and disseminators should be thoughtful about which dissemination approaches are being compared. Greenhalgh (6) suggests that the greatest knowledge gains will come from comparing incremental changes in a proven dissemination approach to test the effect of the changes. She contrasts this incremental testing with all-or-none testing, in which it is often difficult to discern what part of the approach led to significant changes in adoption and implementation. Researchers should also carefully consider study design. Experimental designs are theoretically possible, but dissemination approaches must often be tailored to subgroups of organizations or even individual organizations (as our framework emphasizes), making it difficult to have a standard, replicable approach that can be tested by using an experimental design. Quasi-experimental designs may be more practical.

### Conclusion

We have proposed a practical framework for designing and testing dissemination approaches for evidence-based practices. The framework outlines complementary roles for researchers, disseminators, and user organizations in disseminating evidence-based practices broadly. We have used the framework to disseminate evidence-based promotions for older adult physical activity and workplace health programs nationally. Other researchers and practitioners may find the framework useful in increasing the adoption of evidence-based practices.
